# Undeliverable Sapien 3 prosthesis in transaortic transcatheter aortic valve implantation

**DOI:** 10.1093/jscr/rjab001

**Published:** 2021-02-18

**Authors:** Tomonori Shirasaka, Shingo Kunioka, Yuya Kitani, Hiroyuki Kamiya

**Affiliations:** Department of Cardiac Surgery, Asahikawa Medical University, Asahikawa, Japan; Department of Cardiac Surgery, Asahikawa Medical University, Asahikawa, Japan; Department of Cardiology, Asahikawa Medical University, Asahikawa, Japan; Department of Cardiac Surgery, Asahikawa Medical University, Asahikawa, Japan

## Abstract

In transaortic (TAo) trasncatheter aortic valve implantation (TAVI), direct transmission of forces to the stenotic aortic valve is possible. Therefore, the need of balloon aortic valvoplasty in TAo-TAVI may be very limited regarding the deliverability of TAVI prosthesis. However, if the TAVI prosthesis becomes undeliverable, it becomes seriously problematic. Herein, we present a case of TAo-TAVI in which the TAVI prosthesis was undeliverable, and it was forcefully pushed together with the introducer system into the aortic valve as a bailout technique.

## INTRODUCTION

Balloon aortic valvoplasty (BAV) predilation is no longer considered mandatory for transcatheter aortic valve implantation (TAVI). Currently, BAV is performed only in selected patients [1]. With transaortic TAVI (TAo-TAVI), direct transmission of forces to the stenotic aortic valve is possible, and therefore, the need for BAV in TAo-TAVI may be very limited regarding deliverability of TAVI prosthesis [2]. However, if the TAVI prosthesis becomes undeliverable, it becomes seriously problematic. Here we present a case of TAo-TAVI in which the TAVI prosthesis was undeliverable and was pushed forcefully together with the introducer system into the aortic valve as a bailout technique.

## CASE REPORT

**Figure 1:**
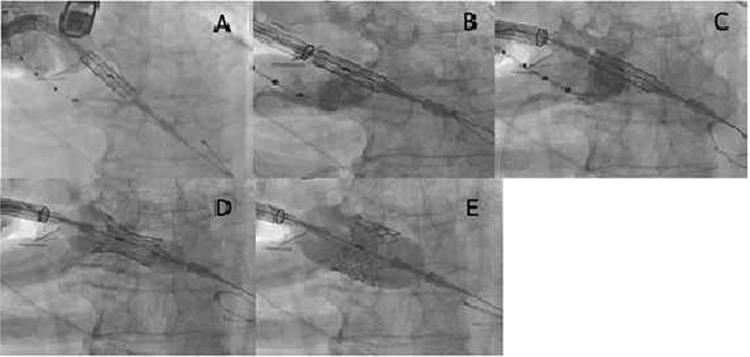
(**A**) The Sapien S3 did not pass into the aortic valve, and the Certitude system bounced back toward reverse direction; (**B**) the Sapien S3 was forcefully pushed together with the Certitude system, and the Sapien S3 finally came into the aortic valve; (**C**) the Certitude system was pulled back to enable dilatation; (**D**) the Sapien S3 was slowly deployed to avoid malposition; (**E**) the Sapien S3 was completely deployed

A male octogenarian patient with symptomatic severe aortic valve stenosis underwent TAo-TAVI because he had highly stenotic common iliac arteries. A right third intercostal surgical incision was made and an 18-Fr introducer sheath was placed at the middle of the ascending aorta. The Certitude delivery system with a 26-mm Sapien S3 transcatheter aortic valve (Edwards Lifesciences, Irvine, CA) was advanced over an extra-small Safari wire (Boston Scientific, Marlborough, MA). However, the Sapien S3 was not able to pass through the aortic valve owing to severe aortic valve stenosis (Fig. 1A). Multiple attempts to pass the valve prosthesis did not work. Because the valve prosthesis was already released from the loader, it could not be brought back into the Certitude system. In this situation, we considered two options: (i) to remove the Sapien S3 together with the Certitude system and restart TAo-TAVI using another system, or (ii) to insert another sheath into the ascending aorta to perform BAV. However, the first option would have been expensive, and the second seemed to be dangerous because the working space through the mini-thoracotomy was very limited.

We then decided on a third option of pushing the Sapien S3 forcefully together with the Certitude system (Fig. 1B). With careful pulling, enough countertraction was given to the Safari wire to ensure the direct transmission of power to the Sapien S3 while simultaneously avoiding injury to the left ventricle by the Safari wire. The Sapien S3 was successfully brought into the aortic valve. Thereafter, the Certitude system was pulled back to near the aortic wall (Fig. 1C), and the Sapien S3 could be deployed as usual (Fig. 1D and E). The postoperative course was uneventful and the patient was discharged home on the 10th postoperativeday.

## DISCUSSION

Compared to conventional transfemoral or transapical TAVI, there has not been so much review in relation to technical tips of Tao-TAVI possibly because this technique has not been widely adopted and the associated results have not been widely reported [3].

When performing TAo TAVI, the angle of insertion of the delivery system into the ascending aorta is crucial and this issue is closely related to where the skin incision is set. In this case, the right third intercostal space as insertion point was selected, which made the insertion angle more verticalized than it had been expected, and therefore the delivery of the Certitude system was not so smooth, and the length of Certitude system in the ascending aorta was too short to put Sapien S3 onto an optimal location antegradely.

## CONCLUSION

We presented a case in which a bailout technique was successfully used in a case of TAo-TAVI in which the TAVI prosthesis was undeliverable. Forced pushing of the Sapien S3 together with the Certitude system into the aortic valve is a feasible option.

## CONFLICT OF INTEREST STATEMENT

None declared.
